# Reconstructive surgery for giant penoscrotal elephantiasis: about one case

**DOI:** 10.1186/2051-4190-24-16

**Published:** 2014-11-17

**Authors:** Brahima Kirakoya, Barnabé Zango, Abdoul Karim Paré, Aristide Fasnéwendé Kaboré, Clotaire Yaméogo

**Affiliations:** Department of Urology Andrology, Yalgado Ouedraogo Teaching Hospital, Ouagadougou, Burkina Faso; Department of Urology Andrology, Hubert koutoucou Maga Teaching Hospital, Cotonou, Bénin

**Keywords:** Elephantiasis, Penoscrotal, Scrotoplasty, Éléphantiasis, Péno scrotal, Scrotoplastie

## Abstract

Elephantiasis of the external genitalia is characterized by lymphedema and thickening of the subcutaneous tissues. This gives the skin an appearance similar to a pachyderm skin. This pathology is invalidating for the patient. Reconstructive surgery is often the only way to restaure aesthetic and functional aspects of the external genitalia. We aim to report a 52 year man with huge penoscrotal elephantiasis who underwent excision and penoscrotal reconstruction at the department of Urology, Yalgado Ouedraogo Teaching Hospital at Ouagadougou.

## Résumé

L’éléphantiasis des organes génitaux externes est caractérisé par un lymphodème et un épaississement des téguments responsable de l’aspect de peau de pachyderme. C’est une pathologie invalidante. La chirurgie de reconstruction constitue souvent la seule alternative pour restaurer l’aspect esthétique et fonctionnel de ces organes atteints. Nous rapportons un cas d’un volumineux éléphantiasis des organes génitaux externes chez un sujet de 52 ans qui a bénéficié avec succès d’une plastie péno scrotale dans le service d’urologie du CHU/YO.

## Introduction

Penoscrotal elephantiasis can lead to hudge enlargement of the scrotal sac and the penis. It is as a result of lymphatic obstruction and subsequent infiltration of the subcutaneous tissue of the external genitalia with lymph [1]. The causes are multiple. It cripple both psychological and physical well-being of the patient. It tends to become an exotic entity even in countries south of the Sahara [2, 3]. The main stay of treatment is surgery.

We aim to report a case of penoscrotal elephantiasis which was managed at the Department of Urology, Yalgado Ouedraogo Teaching Hospital at Ouagadougou (Burkina Faso).

### Observation

**Figure 1:**
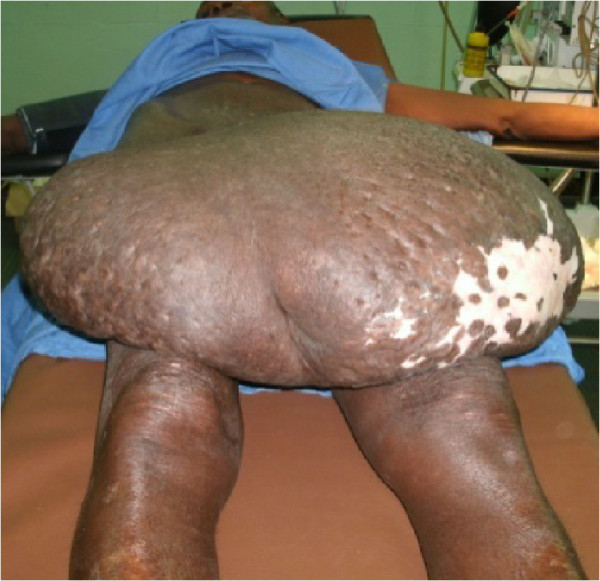
**Scrotal mass.**

A 52 year old cultivator, presented to the department of Urology, Yalgado Ouedraogo Teaching Hospital with history of gradual enlargement scrotal sac over twelve years. The swelling was painless and itchy. No history of a lower urinary tract symptoms, pelvic surgery or venereal disease. Physical examination revealed a hudge scrotal sac extending down to the upper third of the legs (Figure 1). The scrotal skin show many scratch marks and hypopigmented areas. The penile sharp is buried within the scrotal mass. This made it difficult to examine the penis. Urine comes out through a tunnel leading to the glans penis. Testicles could not be palpated due to hudge nature of the scrotal mass. The rest of the physical exam was normal.

**Figure 2:**
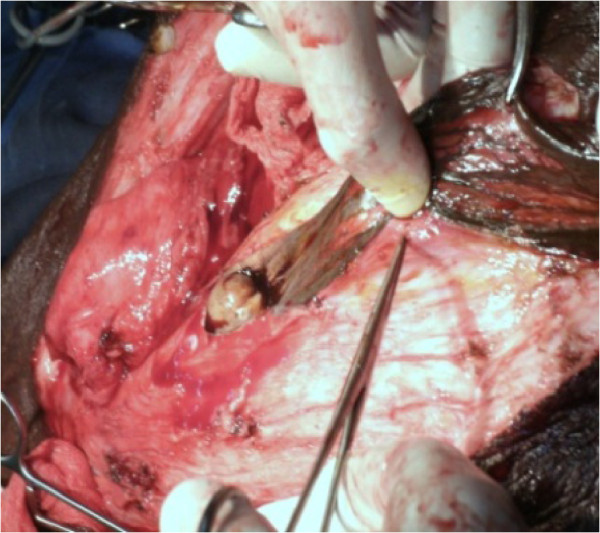
**Anterior midline incision in the scrotal mass.**

Looking at the epidemiological and clinical findings, we made a diagnosis of penoscrotal elephantiasis. The patient had taken a dose of anti filarial drugs (albendazol and ivermectin). He washed the scrotal mass twice per day for three days with a polividon solution. Preoperative testing was performed before surgical procedure. He underwent surgical excision of the subcutaneous tissues and reconstruction of his penis and his scrotum. The procedure was performed as follows:

- Anterior midline incision was made over the scrotal mass, this delivered the penis. The skin of the inner side of the tunnel was found to be normal and was preserve to cover the penis (Figure 2).

**Figure 3:**
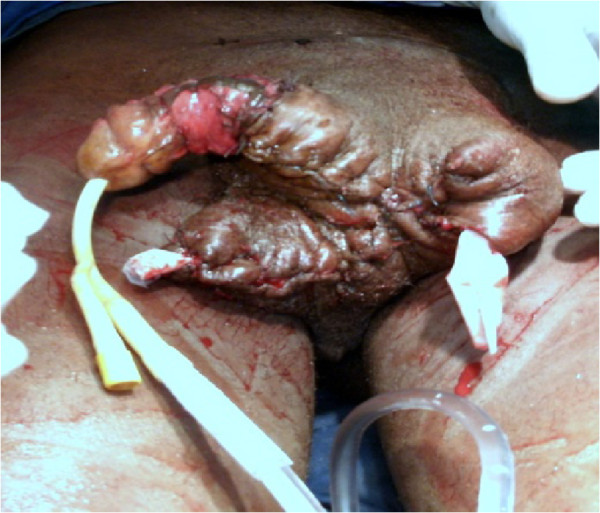
**Postoperative appearance of the external genitalia.**

- Two oblique incisions were made toward the groin starting from cranial end of the previous incision, to find and dissect the spermatid cords and testicles.
- Excision of subcutaneous tissue with careful haemostasis.
- Resection of all abnormal tissue covering the penis.
- Fixation of the testicles to the bottom of the skin flaps to be used for scrotal reconstruction.
- Excision of excess skin.Drainage of both hemi-scrotum which were closed separately using absorbable sutures (Figure 3).
- Bladder was drained with size 20 F foley’s urethral catheter.

The excised scrotal mass contained gelatinous liquid. The subcutaneous tissue was very thick and whitish. All resected tissue weighed 12 kg.

## Result

**Figure 4:**
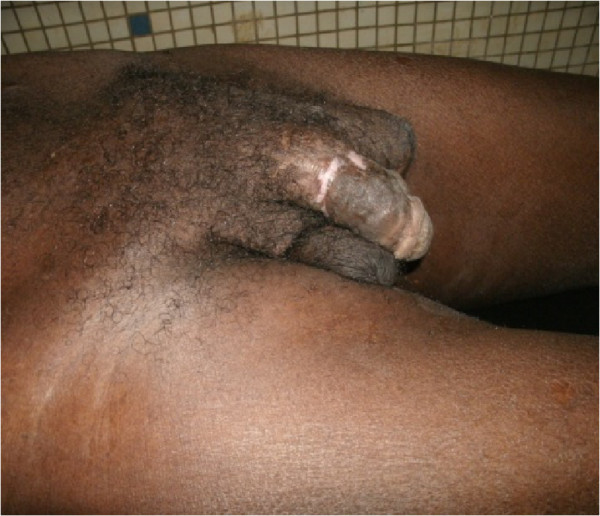
**Appearance of the external genitalia after 3 months.**

The postoperative outcomes were marked by anaemia, which was corrected by compatible blood transfusion. The drains were removed on the fourth post-operative day. No further complication was noted. The penis and the scrotum were cosmetically acceptable at 3 weeks and 5 months post-operative review. The scrotal sac was much reduced and flexible (Figure 4).

## Discussion

The penoscrotal elephantiasis can be defined as massive enlargement of the scrotal sac secondary to a subcutaneous accumulation of lymphatic fluid. It is also called lymphedema [1, 2]. The essential findings to make diagnosis were the appearance of pachyderm skin. It has a debilitating effect on both physical and psychological well-being of the patient. Fortunately the incidence has greatly reduced. It is responsible for 2.1% of giant scrotal sac in Mali [3], while in Senegal N’doye et al. [4] reported an annual incidence of 2 cases.

In tropical countries, microfilaria is the leading cause of elephantiasis of the male external genitalia [5]. The diagnosis is mostly made based on epidemiological evidence and clinical features especially in the early phase of the disease.

Though the diagnosis is made clinically, some laboratory test may be of importance. The microfilariae may be found in the serum, ultrasonography of scrotal sac may pick the adult worms and isotope bi pedal lymphography or radiography may be needed to outline the lymphatics [1]. The aetiology of penoscrotal elephantiasis can be divided into two groups:

- Primary or congenital elephantiasis. In these cases, there is a congenital abnormality of the lymphatic system leading to lymphatics obstruction. It may manifest early or later (Meige disease) in life.
- Secondary elephantiasis. These may be caused by parasitic infection (lymphatic filariasis), bacterial infection, urethral stenosis, pelvic tumor or radiotherapy [6–10]. Treatment may consist of antibiotics especially in the acute phase before the formation of irreversible injuries to the tissue [6, 7, 10]. Delay presentations seem to be common as reported by several African authors [2, 4, 5]. The main stay of treatment of chronic elephantiasis which is associated with irreversible subcutaneous damage remains surgery. Surgical techniques can be divided into two groups.
- Conserving surgery: this is a lymphangioplasty in order to improve lymphatic drainage. These techniques have been abandoned due contradicting results [11].
- Surgical excision: its principle is complete removal of all affected tissue (total lymphangiectomy) and penoscrotoplasty. Reconstructive techniques vary [1, 4, 5]. Many used skin graft with its attendant risk of impaired spermatogenesis due to a change in testicular temperature. Others used scrapped healthy part of the scrotum [1]. We have used this technique for the reconstruction of neoscrotal sac. We are inspired by the technique described by Ouzilleau [12] but with a slight modification on scrotoplasty and the use of a skin flap to cover the penis. In uncircumcised patients, the foreskin can be used to cover the distal part of the penile sharp [13]. The surgical excision of the mass and scrotoplasty provides good results [13]. Complications of this technique include haemorrhage iatrogenic urethral injury, hematoma and surgical site infection. We transfuse after surgery due to primary and reactionary haemorrhage. Incomplete excision may lead to recurrence [11]. Post-operative review revealed satisfactory outcome, both functionally and aesthetically.

## Conclusion

External genitalia elephantiasis is a debilitating condition. It causes both psychological and physically distress. When it affects the external genitalia, there may delay in presentation due to private nature of the area. The commonest aetiological factor in the tropics is microfilariasis. Surgical excision followed by a penoscrotoplasty gives excellent result.

### Consent

Written consent was obtained from the patient for publication of this Case report and any accompanying images.
